# Supplementary material for: Can recent evolutionary history promote resilience to environmental change?
Source: Behav Ecol. 2024 Sep 25;35(6):arae074. doi: 10.1093/beheco/arae074 (PMC11486917; doi:10.1093/beheco/arae074)
Supplement: arae074_suppl_Supplementary_Materials [file arae074_suppl_supplementary_materials.docx]

**Supplementary material**


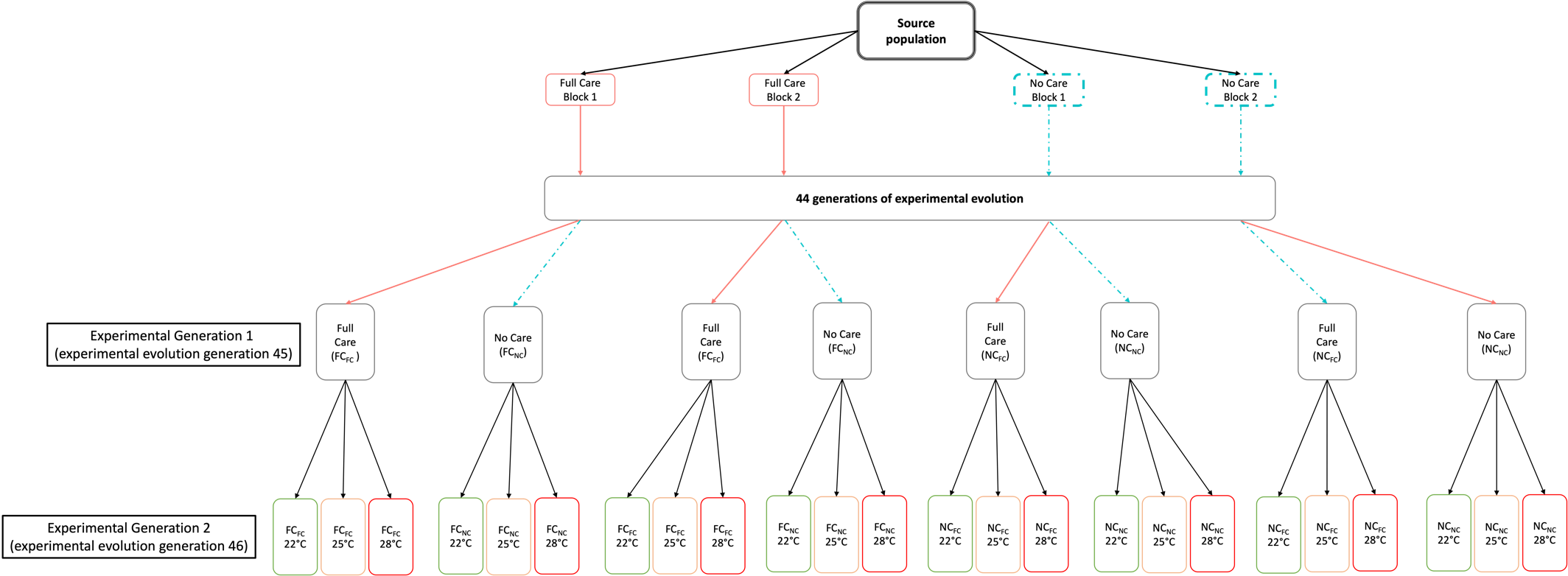


*Figure S1 – Experimental design for breeding protocol*


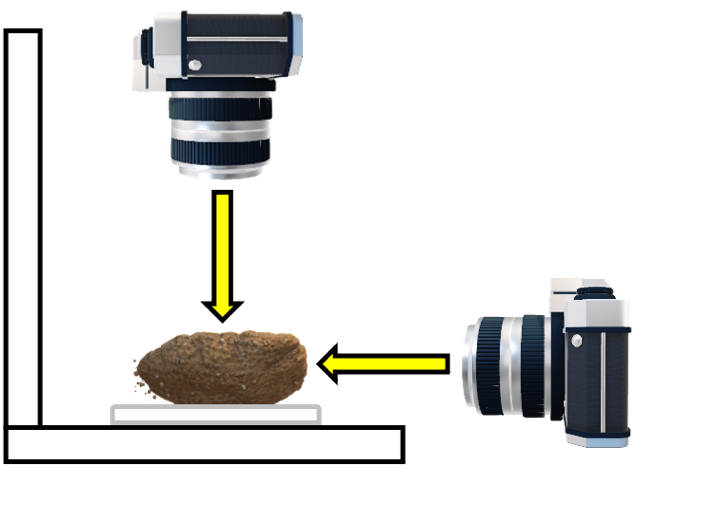


*Figure S2 – Carcass photography protocol (not to scale). Two Canon DSLRs at a 90° angle to each other were used to photograph each carcass from 30 cm away against a white background. The carcass was placed on a clear plastic breeding individual box lid (12 x 8 x 1 cm) to avoid the white background getting contaminated with soil (the lid was wiped between carcasses to maintain a white background for the top image).*

*Script S1 – Carcass roundness script for calculating roundness in ImageJ. The script uses the blue channels from both the top and side photos (as this creates the greatest contrast between the carcass nest and the background), a threshold filter to separate the carcass from the background and a median filter to ignore the smallest black flecks of less than 25 pixels, in case any small soil marks remained.*

//  topPath=File.openDialog("Select top-down image"); // get file location

//  sidePath=File.openDialog("Select side image"); // get file location

Dialog.create("Settings");

Dialog.addMessage("Pixel/distance calibration:");

Dialog.addNumber("Top-down (pixels per mm)", 53.7523);

Dialog.addNumber("Side (pixels per mm)", 30.1806);

Dialog.addMessage("\nImage processing:");

Dialog.addNumber("Threshold", 70);

Dialog.addNumber("Smoothing radius (pixels)", 25);

Dialog.addMessage("\nCamera positioning:");

Dialog.addCheckbox("Flip image", true);

Dialog.show();

topPixelsMm = Dialog.getNumber();

sidePixelsMm = Dialog.getNumber();; thresholdVal = Dialog.getNumber();;; smoothingVal= Dialog.getNumber();;;; flip = Dialog.getCheckbox();

// FILE LOCATIONS

topDir=getDirectory("Directory containing top-down images");

topFileList=getFileList(topDir);

sideDir=getDirectory("Directory containing side-on images");

sideFileList=getFileList(sideDir);

// select working directory // list of images in directory

// select working directory // list of images in directory

if(topFileList.length > sideFileList.length) // error if there are unequal numbers of files exit("Error: unequal number of files in selected folders");

if(topFileList.length < sideFileList.length)

exit("Error: unequal number of files in selected folders");

print("\n________________________________");

print("SETTINGS");

print(" Threshold: " + thresholdVal);

print(" Smoothing Radius: " + smoothingVal);

// START OF BATCH PROCESSING LOOP

for(z=0; z<topFileList.length; z++1){ topPath = topDir+topFileList[z];

sidePath = sideDir+sideFileList[z];

// OPEN & PROCESS TOP IMAGE

open(topPath);

run("RGB Stack");

run("Delete Slice"); // only uses the blue channel (channel with highest contrast)

run("Delete Slice");

setTool(0); //Rectangle tool

waitForUser("Select the Target", "Draw a box to cover the mouse ball\nensure no other dark objects are selected");

run("Crop");

if(flip==1); // if ticked, flips image so that both are the same way round

run("Flip Horizontally");

setThreshold(0, thresholdVal);

run("Convert to Mask");

medianScript = "radius="+smoothingVal+" slice";

run("Median...", medianScript); // threshold

w = getWidth(); // image dimensions

h = getHeight();

// SELECT CENTRE

doWand(w/2, h/2, 1, "4-connected");

List.setMeasurements; // save measurements of perimeter & area

topPerim = List.getValue("Perim.");

topArea = List.getValue("Area");

print("Top Perimeter: " + topPerim + " Top Area: " + topArea);

run("Make Inverse"); // invert selection & delete (gets rid of dirt spots etc..)

setForegroundColor(255, 255, 255);

run("Fill", "slice");

run("Make Inverse");

run("To Bounding Box");

run("Crop");

w = getWidth(); // image dimensions

h = getHeight();

topProfile = newArray(w);

for (x=0; x<w; x++){ // pixel column loop

topProfile[x] = 0;

for (y=0; y<h; y++){ // counts the number of white pixels in each column

} }

val = getPixel(x,y);

if(val==255)

topProfile[x] = topProfile[x] + 1;

topProfileCrop = newArray(0);

for(a=0; a<topProfile.length; a++){ if(topProfile[a]>0){

topProfileCrop = Array.concat(topProfileCrop, topProfile[a]);

} }

topProfileMm = newArray(topProfileCrop.length);

for(a=0; a<topProfileCrop.length; a++){

topProfileMm[a] = topProfileCrop[a] / topPixelsMm;

setResult("Top Pixels", a, topProfileCrop[a]);

setResult("Top Millimetres", a, topProfileMm[a]); }

close();

// OPEN & PROCESS SIDE IMAGE

// make array of millimetre values

open(sidePath);

run("RGB Stack");

run("Delete Slice"); // only uses the blue channel (channel with highest contrast)

run("Delete Slice");

setTool(0); //Rectangle tool

waitForUser("Select the Target", "Draw a box to cover the mouse ball\nensure no other dark objects are selected");

run("Crop");

setThreshold(0, thresholdVal);

run("Convert to Mask");

medianScript = "radius="+smoothingVal+" slice";

run("Median...", medianScript); // threshold

w = getWidth(); // image dimensions

h = getHeight();

// SELECT CENTRE

doWand(w/2, h/2, 1, "4-connected");

List.setMeasurements; // save measurements of perimeter & area

sidePerim = List.getValue("Perim.");

sideArea = List.getValue("Area");

print("Side Perimeter: " + sidePerim + " Side Area: " + sideArea);

run("Make Inverse"); // invert selection & delete (gets rid of dirt spots etc..)

setForegroundColor(255, 255, 255);

run("Fill", "slice");

run("Make Inverse");

run("To Bounding Box");

run("Crop");

w = getWidth(); // image dimensions

h = getHeight();

sideProfile = newArray(w);

for (x=0; x<w; x++){ // pixel column loop

sideProfile[x] = 0;

for (y=0; y<h; y++){ // counts the number of white pixels in each column

} }

val = getPixel(x,y);

if(val==255)

sideProfile[x] = sideProfile[x] + 1;

sideProfileCrop = newArray(0);

for(a=0; a<sideProfile.length; a++){ if(sideProfile[a]>0){

sideProfileCrop = Array.concat(sideProfileCrop, sideProfile[a]);

} }

sideProfileMm = newArray(sideProfileCrop.length);

for(a=0; a<sideProfileCrop.length; a++){

sideProfileMm[a] = sideProfileCrop[a] / sidePixelsMm;

setResult("Side Pixels", a, sideProfileCrop[a]);

setResult("Side Millimetres", a, sideProfileMm[a]); }

// make array of millimetre values

close();

// CIRCUMFRENCE CALCULATION

arrayRatios = topProfileMm.length / sideProfileMm.length;

circumfrenceArray = newArray(sideProfileMm.length);

areaArray = newArray(sideProfileMm.length);

for(i=0; i<sideProfileMm.length; i++){

bLoc = round(arrayRatios*i);

b = topProfileMm[bLoc]/2;

a = sideProfileMm[i]/2;

circumfrenceArray[i] = PI * (a+b) * (1+(3*pow((a-b)/(a+b),2) / (10+pow(4-(3*pow((a- b)/(a+b),2)),0.5))));

areaArray[i] = PI * a * b;

}

topLength = topProfileMm.length / topPixelsMm;

sideLength = sideProfileMm.length / sidePixelsMm;

aveLength = (topLength + sideLength)/2;

mmPerSlice = aveLength/sideProfileMm.length; // average length of mouse

VolumeSum = 0;

AreaSum = 0;

for(a=0; a<sideProfileCrop.length; a++){ // loop to count the area and volume of each slice

AreaSum = AreaSum + (circumfrenceArray[a]*mmPerSlice);

VolumeSum = VolumeSum + (areaArray[a]*mmPerSlice);

// setResult("Circumfrence", a, circumfrenceArray[a]);

}

EndArea = 0;

for(a=0; a<sideProfileCrop.length-1; a++)

EndArea = EndArea + (pow(pow(areaArray[a]-areaArray[a+1],2),0.5)); // sums end surface values - always positive

AreaSum = AreaSum + EndArea;

// SPHERICITY CALCULATION - this takes the average value of the area to circumference ratio of the top & side images

topCircleArea = PI*pow((topPerim/(2*PI)),2); // this calculates the area a circle would have of the observed perimeter length

sideCircleArea = PI*pow((sidePerim/(2*PI)),2);

topRoundness = topArea / topCircleArea; // perfect circle area /observed area, so a perfect circle would be 1, going down to zero with worse roundness

sideRoundness = sideArea / sideCircleArea;

sphericity = (topRoundness+sideRoundness)/2;

// PRINT RESULTS

print("\n...............................................................");

print("Top-down image: " + topFileList[z]);

print("Side-on image: " + sideFileList[z]);

print("Sphericity:\t"+ sphericity);

print("Volume (mm^3):\t"+VolumeSum);

} //end batch processing loop

// average roundness


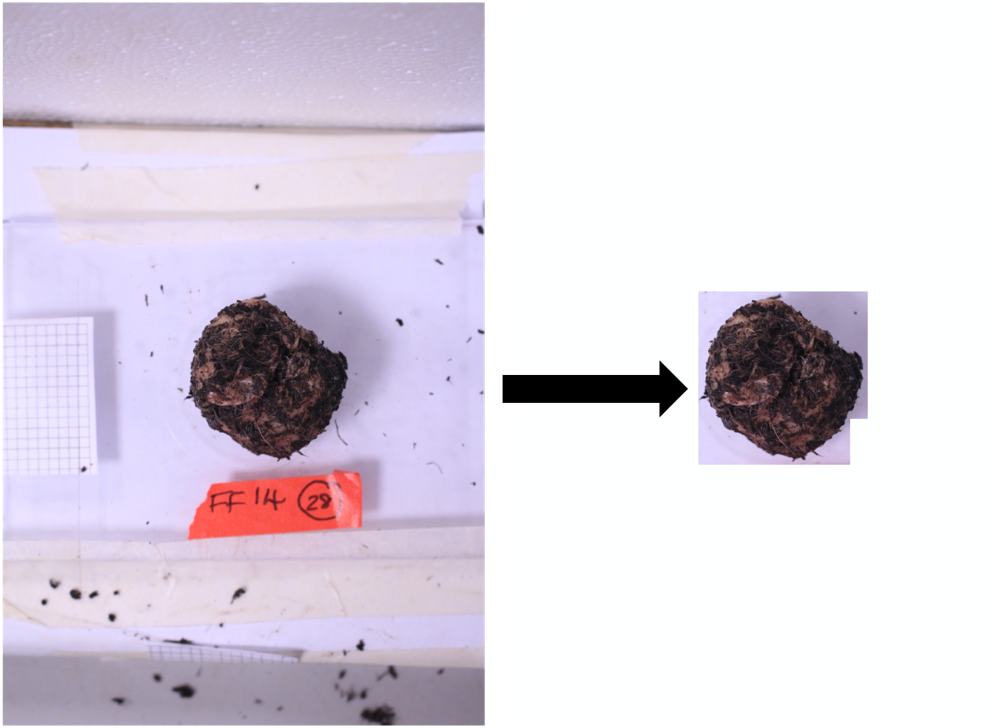


*Figure S3 – The process of removing any dark background marks and identity labels, while ensuring that the size and proportion of the carcass ball remained the same, by digitally superimposing white rectangles over any dark objects in Apple Preview. Script S1 removes black flecks of less than 25 pixels but any larger dark shapes, such as clumps of soil, identity labels and unfurled tails, can influence the carcass roundness calculations so these were digitally removed using this process.*


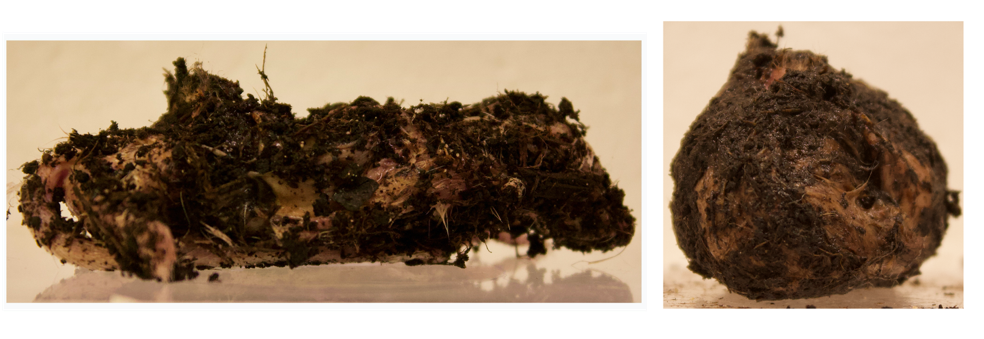


*Figure S4 – An example of the variation in roundness between carcasses. The left image has a roundness value of 0.2957 and the right image has a value of 0.6031. In Script S1 both the top and side images were used to calculate roundness.*

*Table S1 – Interactions included as independent variables in models and the biological reason for their inclusion.*

| **Interaction** | **Reason for inclusion in models** |
| --- | --- |
| Population evolutionary history:temperature | If retained, it indicates that individuals with divergent evolutionary histories have different levels of resilience to thermal stress. This is a central question of the study. |
| Generation 1 care: temperature | If retained, it indicates that individuals that received divergent levels of parental care as larvae have different levels of resilience to thermal stress. This is a central question of the study. |
| Population evolutionary history:generation 1 care | If retained, it indicates that there is an effect that is due to the combination of population’s evolutionary history of care and the care that an individual from one of the populations received as a larva. We could anticipate a scenario in which individuals from a population that evolved with full post-hatching care receives no post-hatching care in generation 1 and, because it is not adapted to a lack of care, suffers fitness consequences which have a subsequent effect on the metrics tested in this study. |
| Population evolutionary history:block | If retained, it indicates that there is an individual population effect, rather than an effect due to the population’s evolutionary history of care or the block in which it was bred. |
| Generation 1 care:block | If retained, it indicates that individuals in different breeding blocks react differently when they receive divergent levels of parental care as larvae. |
| Temperature:block | If retained, it indicates that individuals in different breeding blocks react differently to different levels of thermal stress. This directly investigates the effect of block alone on vulnerability to thermal stress. |
| Population evolutionary history:block:temperature | If retained, it indicates that individual populations (rather than different blocks or evolutionary parental care regimes) react differently to thermal stress. |
| Generation 1 care:block:temperature | If retained, it indicates that individuals in different breeding blocks react differently to thermal stress depending on the care they received as larvae. |

*Table S2 – Model selection for binomial linear regressions of survival in incubators. Models are ordered by AICc, with only models within two AICc points of the model with the lowest AICc shown. The optimal model is in bold. “:” indicates an interaction.*

| **Explanatory variables** | **df** | **Log-likelihood** | **AICc** | **delta** | **weight** |
| --- | --- | --- | --- | --- | --- |
| **block + population evolutionary history + temperature + block:temperature** | **5** | **-277.98** | **566.02** | **0.00** | **0.09** |
| block + generation 1 care + population evolutionary history + temperature + block:temperature | 6 | -277.48 | 567.04 | 1.02 | 0.06 |
| block + generation 1 care + population evolutionary history + temperature + block:temperature + population evolutionary history:generation 1 care | 7 | -276.71 | 567.52 | 1.50 | 0.04 |
| block + population evolutionary history + temperature + block:population evolutionary history + block:temperature | 6 | -277.78 | 567.64 | 1.62 | 0.04 |
| block + population evolutionary history + temperature + block:temperature +population evolutionary history:temperature | 6 | -277.86 | 567.79 | 1.77 | 0.04 |
| block + population evolutionary history + temperature + sex + block:temperature | 6 | -277.93 | 567.92 | 1.90 | 0.04 |
| block + generation 1 care + population evolutionary history + temperature + block:temperature + generation 1 care regime:temperature | 7 | -276.94 | 567.97 | 1.95 | 0.04 |

*Table S3 – Model selection for beta regressions of lytic activity in A) male and B) female anal exudates. Models are ordered by AICc, with only models within two AICc points of the model with the lowest AICc shown. The optimal model is in bold. “:” indicates an interaction.*

| **Explanatory variables** | **df** | **Log-likelihood** | **AICc** | **delta** | **weight** |
| --- | --- | --- | --- | --- | --- |
| 1. **Male exudate** | | | | | |
| block + generation 1 care + carcass mass + temperature | 6 | 110.85 | -208.47 | 0.00 | 0.05 |
| block + generation 1 care + carcass mass | 5 | 109.32 | -207.78 | 0.69 | 0.04 |
| block + generation 1 care + temperature | 5 | 109.29 | -207.72 | 0.75 | 0.04 |
| block + population evolutionary history + generation 1 care + carcass mass + temperature + population evolutionary history:temperature | 8 | 112.76 | -207.35 | 1.12 | 0.03 |
| block + population evolutionary history + generation 1 care + carcass mass + temperature | 7 | 111.42 | -207.17 | 1.29 | 0.03 |
| block + population evolutionary history + generation 1 care + temperature + population evolutionary history:temperature | 7 | 111.32 | -206.98 | 1.49 | 0.03 |
| **block + generation 1 care** | **4** | **107.75** | **-206.92** | **1.55** | **0.02** |
| block + population evolutionary history + generation 1 care + carcass mass | 6 | 109.99 | -206.75 | 1.72 | 0.02 |
| block + generation 1 care + carcass mass + temperature + generation 1 care:temperature | 7 | 111.19 | -206.70 | 1.77 | 0.02 |
| block + population evolutionary history + generation 1 care + temperature | 6 | 109.96 | -206.69 | 1.78 | 0.02 |
| 1. **Female exudate** | | | | | |
| **block + temperature** | **4** | **101.33** | **-194.09** | **0.00** | **0.19** |

*Table S4 – Results of beta regressions investigating the effects on male anal exudate lytic activity at 53h after pairing, after removing one datapoint where <55% of* Micrococcus lysodeikticus *remained (for analyses of the full dataset see Table 2). We have defined lytic activity as the proportion of cell degradation of* M. lysodeikticus *as recorded at 450 nm after 60 minutes.*

| **Independent variable** | **Estimate** | **SE** | **z** | ***p*** |
| --- | --- | --- | --- | --- |
| Intercept | 4.054 | 0.269 | 15.057 | <0.001 |
| Block | -1.333 | 0.147 | -9.091 | <0.001 |

*Table S5 – Model selection for beta regressions of lytic activity in male anal exudates, after removing one datapoint where <55% of* Micrococcus lysodeikticus *remained (for analyses of the full dataset see Table S2). Models are ordered by AICc, with only models within two AICc points of the model with the lowest AICc shown. The optimal model is in bold. “:” indicates an interaction.*

| **Explanatory variables** | **df** | **Log-likelihood** | **AICc** | **delta** | **weight** |
| --- | --- | --- | --- | --- | --- |
| block + generation 1 care + carcass mass | 5 | 111.56 | -212.25 | 0.00 | 0.06 |
| block + generation 1 care + carcass mass + temperature | 6 | 112.61 | -211.97 | 0.28 | 0.05 |
| block + generation 1 care | 4 | 110.14 | -211.70 | 0.55 | 0.04 |
| **block** | **3** | **108.98** | **-211.62** | **0.62** | **0.04** |
| block + generation 1 care + temperature | 5 | 111.20 | -211.51 | 0.74 | 0.04 |
| block + carcass mass | 4 | 109.88 | -211.18 | 1.07 | 0.03 |
| block + temperature | 4 | 109.87 | -211.15 | 1.09 | 0.03 |
| block + carcass mass + temperature | 5 | 110.74 | -210.61 | 1.64 | 0.03 |
| block + population evolutionary history + generation 1 care + carcass mass | 6 | 111.90 | -210.55 | 1.70 | 0.02 |

*Table –5 - Model selection for beta regressions of carcass roundness. Models are ordered by AICc, with only models within two AICc points of the model with the lowest AICc shown. The optimal model is in bold. “:” indicates an interaction.*

| **Explanatory variables** | **df** | **Log-likelihood** | **AICc** | **delta** | **weight** |
| --- | --- | --- | --- | --- | --- |
| **generation 1 care + temperature** | **4** | **132.15** | **-255.99** | **0.00** | **0.10** |
| generation 1 care + carcass mass + temperature | 5 | 132.67 | -254.86 | 1.13 | 0.06 |
| block + generation 1 care + temperature | 5 | 132.41 | -254.33 | 1.65 | 0.04 |
| generation 1 care + temperature + generation 1 care:temperature | 5 | 132.38 | -254.28 | 1.71 | 0.04 |

*Table S6 – Model selection for binomial linear regressions of success vs failure of pairs to lay at least one egg. Models are ordered by AICc, with only models within two AICc points of the model with the lowest AICc shown. The optimal model is in bold. “:” indicates an interaction.*

| **Explanatory variables** | **df** | **Log-likelihood** | **AICc** | **delta** | **weight** |
| --- | --- | --- | --- | --- | --- |
| **block + temperature** | **3** | **-95.32** | **196.71** | **0.00** | **0.03** |
| block + population evolutionary history + temperature + block:population evolutionary history | 5 | -93.28 | 196.72 | 0.01 | 0.03 |
| block + population evolutionary history + temperature + block:population evolutionary history + block:temperature | 6 | -92.37 | 196.96 | 0.25 | 0.03 |
| block + temperature + block:temperature | 4 | -94.45 | 197.00 | 0.29 | 0.03 |
| block + population evolutionary history + temperature + population evolutionary history:temperature | 5 | -93.45 | 197.06 | 0.36 | 0.02 |
| block + population evolutionary history + temperature | 4 | -94.57 | 197.25 | 0.55 | 0.02 |
| block + generation 1 care + temperature + generation 1 care:temperature | 5 | -93.59 | 197.34 | 0.63 | 0.02 |
| block + population evolutionary history + generation 1 care + temperature + block:population evolutionary history + generation 1 care:temperature | 7 | -91.52 | 197.34 | 0.64 | 0.02 |
| block + population evolutionary history + temperature + block:temperature + population evolutionary history:temperature | 6 | -92.57 | 197.37 | 0.66 | 0.02 |
| block + population evolutionary history + temperature + block:temperature | 5 | -93.69 | 197.53 | 0.82 | 0.02 |
| block + population evolutionary history + generation 1 care + temperature + block:population evolutionary history + block:temperature + generation 1 care:temperature | 8 | -90.62 | 197.62 | 0.91 | 0.02 |
| block + generation 1 care + temperature + block:temperature + generation 1 care:temperature | 6 | -92.72 | 197.66 | 0.95 | 0.02 |
| block + population evolutionary history + generation 1 care + temperature + population evolutionary history:temperature + generation 1 care:temperature | 7 | -91.71 | 197.72 | 1.01 | 0.02 |
| block + population evolutionary history + generation 1 care + temperature + generation 1 care:temperature | 6 | -92.82 | 197.86 | 1.15 | 0.02 |
| block + population evolutionary history + temperature + block:population evolutionary history + population evolutionary history:temperature | 6 | -92.92 | 198.05 | 1.35 | 0.01 |
| block + population evolutionary history + generation 1 care + temperature + block:temperature + population evolutionary history:temperature + generation 1 care:temperature | 8 | -90.84 | 198.06 | 1.35 | 0.01 |
| block + population evolutionary history + generation 1 care + temperature + block:temperature + generation 1 care:temperature | 7 | -91.93 | 198.16 | 1.46 | 0.01 |
| block + population evolutionary history + carcass mass + temperature + block:population evolutionary history | 6 | -93.13 | 198.48 | 1.78 | 0.01 |
| block + population evolutionary history + temperature + block:population evolutionary history + block:temperature + population evolutionary history:temperature | 7 | -92.10 | 198.50 | 1.80 | 0.01 |
| block + carcass mass + temperature | 4 | -95.20 | 198.51 | 1.80 | 0.01 |
| block + population evolutionary history + carcass mass + temperature + block:population evolutionary history + block:temperature | 7 | -92.19 | 198.68 | 1.97 | 0.01 |
| block + population evolutionary history + generation 1 care + temperature + block:population evolutionary history + population evolutionary history:temperature + generation 1 care:temperature | 8 | -91.16 | 198.70 | 2.00 | 0.01 |

*Table S8 – Model selection for quasipoisson linear regressions of clutch size. Models are ordered by AICc, with only models within two AICc points of the model with the lowest AICc shown. The optimal model is in bold. “:” indicates an interaction.*

| **Explanatory variables** | **df** | **Log-likelihood** | **AICc** | **delta** | **weight** |
| --- | --- | --- | --- | --- | --- |
| **block + temperature + block:temperature** | **5** | **-1301.09** | **718.83** | **0.00** | **0.05** |
| block + generation 1 care + temperature + block:temperature | 6 | -1298.16 | 719.24 | 0.41 | 0.04 |
| block + generation 1 care + temperature + block:generation 1 care + block:temperature | 7 | -1294.84 | 719.44 | 0.60 | 0.04 |
| block + carcass mass + temperature + block:temperature | 6 | -1299.61 | 720.03 | 1.20 | 0.03 |
| block + population evolutionary history + temperature + block:temperature + population evolutionary history:temperature | 7 | -1296.63 | 720.41 | 1.58 | 0.02 |
| block + generation 1 care + carcass mass + temperature + block:temperature | 7 | -1296.71 | 720.45 | 1.62 | 0.02 |
| block + generation 1 care + temperature + block:temperature + generation 1 care:temperature | 7 | -1297.07 | 720.65 | 1.82 | 0.02 |
| block + generation 1 care + carcass mass + temperature + block:generation 1 care + block:temperature | 8 | -1293.56 | 720.75 | 1.91 | 0.02 |
| block + population evolutionary history + temperature + block:temperature | 6 | -1300.99 | 720.78 | 1.95 | 0.02 |

*Table S9 – Model selection for binomial linear regressions of brood hatching success vs failure. Models are ordered by AICc, with only models within two AICc points of the model with the lowest AICc shown. The optimal model is in bold. “:” indicates an interaction.*

| **Explanatory variables** | **df** | **Log-likelihood** | **AICc** | **delta** | **weight** |
| --- | --- | --- | --- | --- | --- |
| block + carcass mass + temperature | 4 | -100.93 | 209.97 | 0.00 | 0.02 |
| block + temperature | 3 | -102.00 | 210.07 | 0.10 | 0.02 |
| carcass mass + temperature | 3 | -102.14 | 210.34 | 0.36 | 0.02 |
| block + population evolutionary history + carcass mass + temperature + block:population evolutionary history | 6 | -99.14 | 210.50 | 0.53 | 0.02 |
| block + population evolutionary history + temperature + block:population evolutionary history | 5 | -100.22 | 210.61 | 0.64 | 0.02 |
| block + carcass mass + temperature + block:temperature | 5 | -100.30 | 210.75 | 0.78 | 0.02 |
| block + temperature + block:temperature | 4 | -101.35 | 210.80 | 0.83 | 0.02 |
| **temperature** | **2** | **-103.47** | **210.98** | **1.01** | **0.01** |
| block + generation 1 care + carcass mass + temperature | 5 | -100.46 | 211.08 | 1.11 | 0.01 |
| block + population evolutionary history + carcass mass + temperature + block:population evolutionary history + temperature:population evolutionary history | 7 | -98.40 | 211.10 | 1.13 | 0.01 |
| block + population evolutionary history + temperature + block:population evolutionary history + temperature:population evolutionary history | 6 | -99.48 | 211.18 | 1.21 | 0.01 |
| block + generation 1 care + temperature | 4 | -101.57 | 211.25 | 1.28 | 0.01 |
| block + population evolutionary history + carcass mass + temperature + block:population evolutionary history + block:temperature | 7 | -98.54 | 211.38 | 1.41 | 0.01 |
| block + population evolutionary history + temperature + block:population evolutionary history + block:temperature | 6 | -99.61 | 211.44 | 1.46 | 0.01 |
| generation 1 care + carcass mass + temperature | 4 | -101.68 | 211.47 | 1.50 | 0.01 |
| block + population evolutionary history + carcass mass + temperature | 5 | -100.69 | 211.54 | 1.57 | 0.01 |
| block + population evolutionary history + temperature | 4 | -101.72 | 211.54 | 1.57 | 0.01 |
| block + generation 1 care + carcass mass + temperature + block:generation 1 care | 6 | -99.69 | 211.61 | 1.64 | 0.01 |
| block + population evolutionary history + generation 1 care + carcass mass + temperature + block:population evolutionary history | 7 | -98.67 | 211.64 | 1.67 | 0.01 |
| block + generation 1 care + carcass mass + temperature + temperature:generation 1 care | 6 | -99.78 | 211.79 | 1.82 | 0.01 |
| block + population evolutionary history + generation 1 care + temperature + block:population evolutionary history | 6 | -99.80 | 211.82 | 1.85 | 0.01 |
| block + generation 1 care + carcass mass + temperature + block:temperature | 6 | -99.82 | 211.87 | 1.90 | 0.01 |
| population evolutionary history + carcass mass + temperature | 4 | -101.89 | 211.89 | 1.92 | 0.01 |
| block + generation 1 care + temperature + block:generation 1 care | 5 | -100.87 | 211.90 | 1.93 | 0.01 |
| block + population evolutionary history + carcass mass + temperature + block:population evolutionary history + block:temperature + temperature:population evolutionary history | 8 | -97.78 | 211.95 | 1.98 | 0.01 |

*Table S10 –* *Model selection for quasipoisson linear regressions of brood size. Models are ordered by AICc, with only models within two AICc points of the model with the lowest AICc shown. The optimal model is in bold. “:” indicates an interaction.*

| **Explanatory variables** | **df** | **Log-likelihood** | **AICc** | **delta** | **weight** |
| --- | --- | --- | --- | --- | --- |
| block + clutch size + carcass mass + temperature | 6 | -845.36 | 409.81 | 0.00 | 0.06 |
| block + clutch size + population evolutionary history + carcass mass + temperature | 7 | -842.31 | 410.38 | 0.57 | 0.05 |
| block + clutch size + carcass mass + temperature + block:temperature | 7 | -842.70 | 410.57 | 0.76 | 0.04 |
| **block + clutch size + temperature** | **5** | **-851.51** | **410.69** | **0.88** | **0.04** |
| block + clutch size + population evolutionary history + carcass mass + temperature + block:temperature | 8 | -839.80 | 411.20 | 1.40 | 0.03 |
| block + clutch size + temperature + block:temperature | 6 | -848.58 | 411.32 | 1.51 | 0.03 |
| block + clutch size + generation 1 care + carcass mass + temperature | 7 | -844.97 | 411.62 | 1.82 | 0.03 |
| block + clutch size + population evolutionary history + carcass mass + temperature + temperature:population evolutionary history | 8 | -840.96 | 411.75 | 1.94 | 0.02 |
| block + clutch size + population evolutionary history + temperature | 6 | -849.64 | 411.81 | 2.00 | 0.02 |
